# A Case of Cutibacterium acnes Pericardial Effusion After Coronary Artery Bypass Surgery

**DOI:** 10.7759/cureus.43095

**Published:** 2023-08-07

**Authors:** Travis R Moodie, John Taylor, Benjamin Shepple

**Affiliations:** 1 Internal Medicine, University of Tennessee, Knoxville, USA; 2 Cardiology, University of Tennessee Medical Center, Knoxville, USA

**Keywords:** coronary artery bypass grafting (cabg), bacterial pericarditis, perioperative echo, echocardiogram (echo), transesophageal echo, loculated pericardial effusion, purulent pericarditis, cutibacterium acnes

## Abstract

*Cutibacterium acnes* is a gram-positive, anaerobic rod commonly found on the skin and mucosal membrane. It is mostly associated with its role in acne formation, but here we present a case of purulent pericarditis secondary to *C. acnes* after coronary artery bypass graft surgery (CABG). A 58-year-old male presented for CABG after a coronary angiogram showed severe multivessel disease. The procedure was performed successfully. He had minimal complications until postop day seven, when he developed a fever and hypoxia. The transthoracic echo (TTE) was largely unrevealing. Due to further declining status the following day, a transesophageal echo (TEE) was performed and revealed a loculated pericardial effusion not visualized on TTE. This was subsequently drained, and fluid cultures grew *C. acnes*. The patient received five weeks of antibiotic therapy, which improved his condition.

## Introduction

*Cutibacterium acnes* is a gram-positive, anaerobic rod commonly found on the skin and mucosal membrane. It is most discussed for its role in acne formation. However, it can be implicated in more serious infections. *Staphylococcus aureus* and *Streptococcus* species are considered more common causes of bacterial pericarditis, but *C. acnes* is increasingly recognized as a causative organism [1-2]. Here, we present the case of *C. acnes* causing purulent pericarditis after coronary artery bypass graft (CABG) surgery.

## Case presentation

Patient information 

A 58-year-old male with a past medical history of poorly controlled type II diabetes, hypertension, hyperlipidemia, and obesity presented with two weeks of substernal chest pain that radiated to the left shoulder and jaw. His electrocardiogram (EKG) in the outpatient setting showed normal sinus rhythm without significant ST changes. He was sent for a stress test EKG that showed a 2 mm horizontal ST segment depression in leads II, III, AVF, V5, and V6 consistent with ischemia. He was admitted to the hospital for urgent left heart catheterization (LHC). His LHC showed multivessel coronary artery disease with complete thrombotic occlusion to the mid-right coronary artery (RCA) with left greater than right collaterals, 70% mid to 80% distal left anterior descending (LAD) stenosis, and 80% proximal stenosis. Cardiothoracic surgery was consulted and performed a three-vessel CABG surgery with a left internal mammary artery (LIMA) to the LAD and a reversed saphenous venous graft (RSVG) to marginal and distal RCA. Afterwards, the patient was started on guideline-directed medical therapy with aspirin, clopidogrel, atorvastatin, and metoprolol.

Investigation 

**Figure 1:**
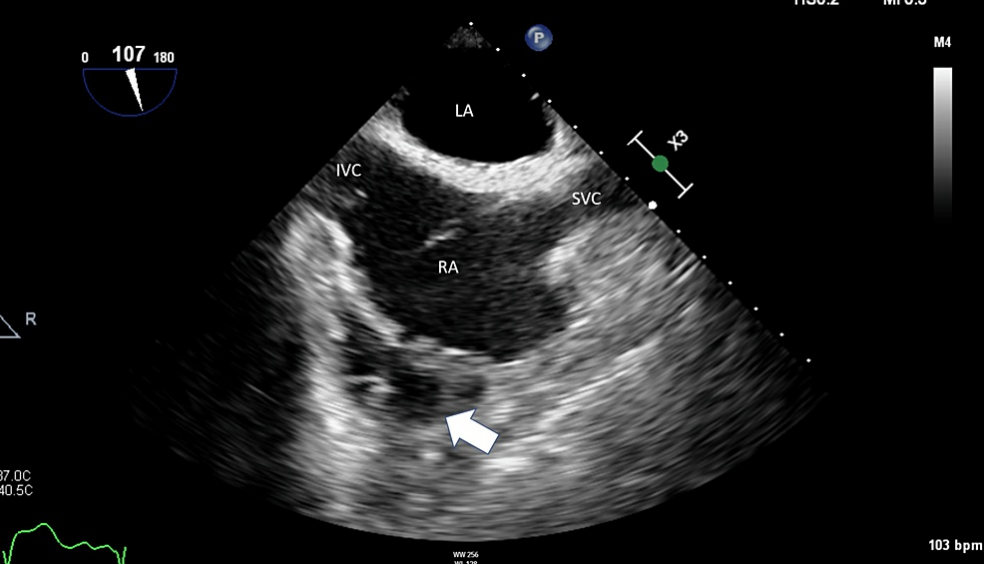
Two-dimensional TEE still frame showing the mid esophageal bicaval view (107°) during mid systole, demonstrating a loculated pericardial effusion (arrow) adjacent to the RA TEE: Transesophageal echo; IVC: Inferior vena cava; LA: Left atrium; RA: Right atrium; SVC: Superior vena cava

**Figure 2:**
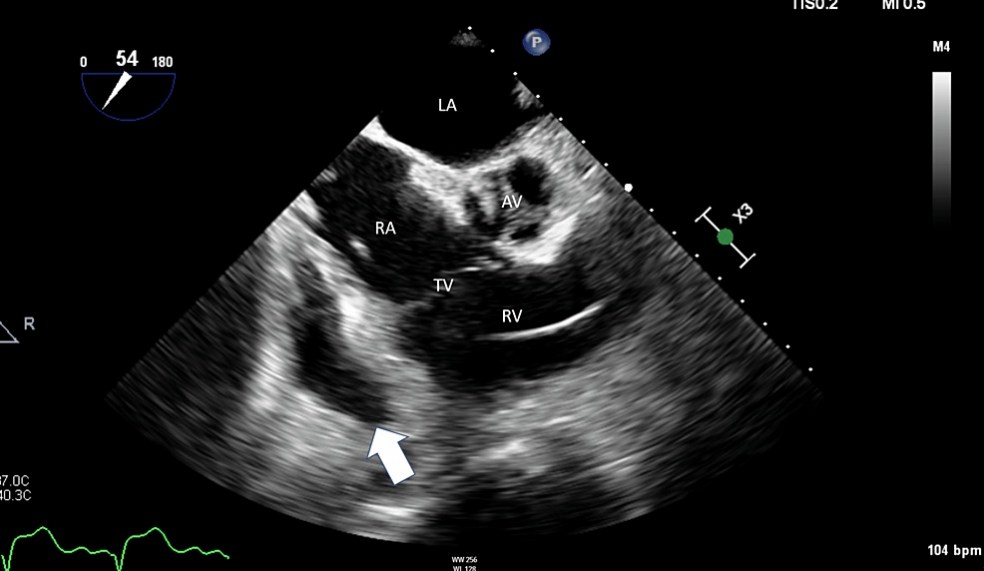
Two-dimensional TEE still frame showing the mid esophageal aortic valve short axis view (54°) during early systole, demonstrating a loculated pericardial effusion (arrow) adjacent to the RA and RV TEE: Transesophageal echo; AV: Aortic valve; LA: Left atrium; RA: Right atrium; RV: Right ventricle; TV: Tricuspid valve

The patient did well for the first week after surgery with complications of postoperative atrial fibrillation managed with amiodarone as well as hypoxia secondary to low cardiac output after the procedure, which was successfully managed with diuretics. On postop day seven, the patient became increasingly hypoxic, developed a significant leukocytosis, and became feverish with a temperature of 100.4 degrees Fahrenheit. Chest X-ray showed interval worsening of bilateral perihilar airspace opacities and new right upper lung airspace opacity with an unchanged cardiac silhouette. A transthoracic echo (TTE) was obtained that showed a low normal left ventricular ejection fraction (LVEF) of 50% to 55%, segmental hypokinesis of the left ventricular apex consistent with LHC findings, poorly visualized but grossly normal right ventricle (RV) size and function, and no pericardial effusion. Blood and sputum cultures were obtained, and the patient was started on linezolid and piperacillin-tazobactam for hospital-acquired pneumonia. On postop day eight, the patient continued to deteriorate. He required intubation for acute hypoxic respiratory failure. Upon intubation, the patient became bradycardic and significantly hypotensive. He went into cardiac arrest, and he required one round of chest compressions and initiation of intravenous epinephrine before resuscitation was achieved. A right heart catheterization showed a pulmonary artery pressure of 50/30 with a central venous pressure of 20 mmHg. A bedside transesophageal (TEE) was performed, showing low normal left ventricular systolic dysfunction, normal RV size and function, no hemodynamically significant valvular disease, and a loculated pericardial effusion on the right atrium and ventricle suggesting right atrial tamponade (Figures 1-2). A bedside cardiac window was completed, draining 300 cc to 400 cc of dark, bloody fluid that was sent for cultures. Anidulafungin was added for fungal coverage in addition to current antibiotics. Reevaluation the following day showed the patient's blood pressure was stable of vasopressor support and his hypoxia had improved. His fever had resolved, and his leukocytosis had improved. Blood and sputum cultures were negative. Wound cultures returned with *C. acnes*. 

Treatment 

Antifungal coverage was discontinued, and antibiotics were switched to unasyn with plans to discharge on penicillin infusions for a minimum of four weeks. Unfortunately, the patient developed *Klebsiella aerogenes *pneumonia and mediastinitis, requiring the broadening of antibiotics to meropenem and vancomycin. The patient had an allergic reaction while on these medications, and he was subsequently placed on levofloxacin for *K. aerogenes* coverage and linezolid for *C. acnes* coverage. He completed a five-week course of antibiotics during his hospital stay before being transferred to a skilled nursing facility for further management. 

Follow-up and outcome 

The patient’s hospital course was further complicated by atrial fibrillation, treated successfully with rate control and sternal dehiscence, requiring sternal rewiring and wound vac placement. Post-hospitalization complications include a protruding sternal wire, further complicated by a *Staphylococcus* infection and treated with sternal wire removal with wound debridement and antibiotics. 

## Discussion

Purulent pericarditis is a rare and potentially life-threatening condition. *Staphylococcus aureus* and *Streptococcus* species are the most common causes, but* C. acnes* is becoming an increasingly recognized cause of bacterial pericarditis [1]. One single-center retrospective 10-year analysis showed a higher incidence of* C. acnes* than both *S. aureus* and *Streptococcus* species [2]. The diagnosis of a pericardial effusion is typically made with TTE. However, effusions after CABG are more likely to be loculated (up to 58% of post-CABG effusions) and located anteriorly [3]. These may be technically challenging to visualize on TTE due to several factors, including surgical wound dressings and hemodynamic monitoring devices [4]. Physicians should maintain a low threshold to pursue additional imaging. Studies have shown TEE to be more sensitive than TTE at detecting pericardial disease, and it has been shown to be safe for critically ill patients, such as ours [4-11]. Cardiac computerized tomography (CCT) is also recommended as an adjunct to TTE in detecting pericardial disease as a quick, more sensitive imaging modality that provides additional information on surrounding structures [5-7,10-11]. A CCT may be used with or without contrast for the diagnosis of pericarditis. Contrast is often preferred for highlighting active pericarditis or tumor infiltration [11]. Drawbacks include radiation exposure and contrast reactions, if utilized. Cardiovascular magnetic resonance imaging is the most sensitive imaging modality for detecting pericardial disease, with detection rates as high as 94% to 100% [7]. It is also considered a secondary imaging modality for pericardial disease as it is time-consuming, expensive, and may have reduced image quality with arrhythmias [5-7,10-11]. 

Imaging modality should be chosen based on the patient's clinical scenario, patient's hemodynamic stability, and the benefits and drawbacks listed above. Patients such as ours with hemodynamic compromise or high suspicion for purulent pericarditis should undergo pericardial drainage with subsequent fluid analysis [1]. *Cutibacterium acnes *is typically susceptible to penicillin, cephalosporin, carbapenems, vancomycin and aminoglycosides [12]. The duration of therapy can be variable. One analysis showed successful treatments ranging from two weeks to over a year in duration [13]. In general, four to six weeks is an appropriate starting point. Additionally, treatment with nonsteroidal anti-inflammatory drugs and colchicine can also be added unless otherwise contraindicated [2,14]. Patients should be monitored for recurrence as well as constrictive pericarditis, as both events have been recorded [14,15]. 

## Conclusions

*Cutibacterium acnes* is becoming an increasingly recognized cause of purulent pericarditis. Here, we presented a case of *C. acnes *causing purulent pericarditis after coronary artery bypass surgery. Diagnosis is typically made with TTE, but further imaging such as TEE, cardiac CT, or cardiac MRI may be necessary if TTE is negative and clinical suspicion remains high. Treatment typically includes drainage and four to six weeks of antibiotics. 

## References

[1] Chiabrando JG, Bonaventura A, Vecchié A (2020). Management of acute and recurrent pericarditis: JACC state-of-the-Art review. J Am Coll Cardiol.

[2] Mookadam F, Moustafa SE, Sun Y (2009). Infectious pericarditis: an experience spanning a decade. Acta Cardiol.

[3] Pepi M, Muratori M, Barbier P (1994). Pericardial effusion after cardiac surgery: incidence, site, size, and haemodynamic consequences. Br Heart J.

[4] Berge K, Lanier LW, Reeder G Occult cardiac tamponade detected by transesophageal echocardiography. Mayo Clin Proc.

[5] Khandaker MH, Espinosa RE, Nishimura RA, Sinak LJ, Hayes SN, Melduni RM, Oh JK (2010). Pericardial disease: diagnosis and management. Mayo Clin Proc.

[6] Adler Y, Charron P, Imazio M (2015). 2015 ESC guidelines for the diagnosis and management of pericardial diseases: The Task Force for the Diagnosis and Management of Pericardial Diseases of the European Society of Cardiology (ESC) endorsed by: The European Association for Cardio-Thoracic Surgery (EACTS). Eur Heart J.

[7] Klein AL, Abbara S, Agler DA (2013). American Society of Echocardiography clinical recommendations for multimodality cardiovascular imaging of patients with pericardial disease: endorsed by the Society for Cardiovascular Magnetic Resonance and Society of Cardiovascular Computed Tomography. J Am Soc Echocardiogr.

[8] Hutchison SJ, Smalling RG, Albornoz M, Colletti P, Tak T, Chandraratna PAN (1994). Comparison of transthoracic and transesophageal echocardiography in clinically overt or suspected pericardial heart disease. Am J Cardiol.

[9] Kochar GS, Jacobs LE, Kotler MN (1990). Right atrial compression in postoperative cardiac patients: detection by transesophageal echocardiography. J Am Coll Cardiol.

[10] Bernard Cosyns, Sven Plein, Petros Nihoyanopoulos (2015). On behalf of the European Association of Cardiovascular Imaging (EACVI) and European Society of Cardiology Working Group (ESC WG) on Myocardial and Pericardial diseases, European Association of Cardiovascular Imaging (EACVI) position paper: multimodality imaging in pericardial disease. Eur Heart J Cardiovasc Imaging.

[11] Yared K, Baggish AL, Picard MH, Hoffmann U, Hung J (2010). Multimodality imaging of pericardial diseases. JACC Cardiovasc Imaging.

[12] Steven RD, Papia K, Subhashis M (2021). Bacterial pericarditis and empyema caused by Cutibacterium acnes in a patient with metastatic lung cancer. Anaerobe.

[13] Li-Geng T, Geraci TC, Narula N (2021). Recognizing Cutibacterium acnes as a cause of infectious pericarditis: a case report and review of literature. Anaerobe.

[14] Fakhri G, Tayeh C, Dbaibo G, El Sedawy O, Abdul Halim N, Bitar F, Arabi M (2020). Cardiac tamponade caused by Cutibacterium acnes: an updated and comprehensive review of the literature. Can J Infect Dis Med Microbiol.

[15] Jensen TB, Kheyr MA, Mohey R (2017). Constrictive pericarditis caused by Cutibacterium (Propionibacterium) acnes: a case report and review of literature. IDCases.

